# Draft Genome Sequence of *Salmonella enterica* subsp. *enterica* Serovar Give, Isolated from an Imported Chili Powder Product

**DOI:** 10.1128/genomeA.00726-15

**Published:** 2015-07-02

**Authors:** Hua Wang, Yi Chen, Sherry Ayers, David Melka, Anna Laasri, Justin S. Payne, Jie Zheng, Insook Son, Ruth Timme, George Kastanis, Thomas S. Hammack, Errol Strain, Marc W. Allard, Peter S. Evans, Eric W. Brown

**Affiliations:** a Division of Microbiology, Center for Food Safety and Applied Nutrition, U.S. Food and Drug Administration, College Park, Maryland, USA; b Division of Animal and Food Microbiology, Center for Veterinary Medicine, U.S. Food and Drug Administration, Laurel, Maryland, USA

## Abstract

We report the genome sequence of *Salmonella enterica* subsp. *enterica* serovar Give (CFSAN012622), isolated from imported chili powder in 2014. This genome contains genes previously reported to be specific only to *S. enterica* serovar Enteritidis. This strain shows a unique pulsed-field gel electrophoresis (PFGE) pattern clustering with serovar Enteritidis (JEG X01.0005).

## GENOME ANNOUNCEMENT

The United States is one of the largest spice importers on the basis of both volume and value (http://www.ers.usda.gov/data-products/food-availability-%28per-capita%29-data-system.aspx), importing more than 80% of its total spice supply (1). Contamination by *Salmonella* was the cause of 95% of the U.S. food recalls associated with spices between the years 1980–2000 (2) and three large-scale salmonellosis outbreaks in the United States attributed to consumption of *Salmonella*-contaminated spices/seasonings between 2007 and 2010 (3, 4) (http://www.cdph.ca.gov/programs/DFDRS/Documents/QSS_Presentation_SRissen_and_%20white%20pepper_010611.pdf). A major FDA investigation into the prevalence of *Salmonella* in dried spices reported that nearly 7% of imported shipments of spice offered for entry to the United States during 2007 to 2009 were contaminated, even though some spices may have been subjected to pathogen reduction treatments (1).

During our recent surveillance of dried spices, the *Salmonella enterica* subsp. *enterica* serovar Give (CFSAN012622) strain was isolated from an imported chili powder product using the Bacteriological Analytical Manual (BAM) *Salmonella* culture method (http://www.fda.gov/Food/FoodScienceResearch/LaboratoryMethods/ucm070149.htm), then serotyped using Luminex *Salmonella* serotyping assay (5–7) and traditional serological methods (8). The serotype antigens were E: l, v: 1, 7. Pulse-field gel electrophoresis (PFGE) using restriction enzyme XbaI was performed according to CDC methods (http://www.cdc.gov/pulsenet/PDF/ecoli-shigella-salmonella-pfge-protocol-508c.pdf). This strain shows a unique PFGE pattern, clustering with *S*. Enteritidis with a PFGE pattern JEG X01.0005. The genomic DNA from CFSAN012622 was isolated from overnight culture using the DNeasy blood and tissue kit (Qiagen, Valencia, CA), and its genome was sequenced using an Illumina MiSeq (Illumina, San Diego, CA) according to the manufacturer’s instructions. Genomic sequence contigs were assembled using SPAdes software version 3.0.0, and the sequence was annotated using the NCBI Prokaryotic Genomes Automatic Annotation Pipeline (9). Genomic comparison was performed using CLC Genomics Workbench 6.0.2 (CLC Bio, Denmark).

The genome for CFSAN012622 is 4,735,685 bp long, containing DNA regions matching *Salmonella* difference fragments (Sdf) regions I, III, and IV, which have previously been reported only in *S*. Enteritidis CAHFS-5 (10). This genome also carries genes *ydaO*, *lygA*, *lygD*, *lygE*, *lygF* in the *Salmonella* difference region I (Sdr I) which had been reported as unique to *S*. Enteritidis CAHFS-285 (10). Genomic comparisons showed that draft genome of CFSAN012622 has a 91.34% match to the draft genome of CFSAN004343 (GenBank accession no. AYUQ00000000), an *S*. Give strain isolated from cow feces with 64,929 single nucleotide polymorphism (SNP) difference. Two genomes of *S*. Enteritidis strains (*S*. Enteritidis strains 13-1 and PT23) which have PFGE pattern JEG X01.0005 clustering with *S*. Give (CFSAN012622) were used for the genome comparison. The draft genome of CFSAN012622 has an 89.32% match to the draft genome of *S*. Enteritidis strain 13-1 (GenBank accession no. AHUQ00000000) with a 61,156 SNP difference. The draft genome of CFSAN012622 has an 89.29% similarity to the draft genome of *S*. Enteritidis strain PT23 (GenBank accession no. AHUR00000000) with a 61,004 SNP difference. Therefore, it is not surprising that the PCR used to specifically detect *S*. Enteritidis targeting Sdf gene generated a positive result for CFSAN012622.

### Nucleotide sequence accession numbers.

This whole-genome shotgun project has been deposited in DDBJ/EMBL/GenBank under the accession no. JOKK00000000. The version described in this paper is the first version, JOKK01000000.
